# Profiling the Athletes’ Gut Microbiome: A Critical Methodological Perspective on 16S Metabarcoding and Shotgun Metagenomics

**DOI:** 10.3390/biology15080600

**Published:** 2026-04-10

**Authors:** Junior Carlone, Ághata Cardoso da Silva Ribeiro, Attilio Parisi, Saverio Giampaoli, Alessio Fasano

**Affiliations:** 1 Department of Neurosciences, Biomedicine and Movement, University of Verona, 37134 Verona, Italy; 2 Department of Movement, Human and Health Sciences, University of Rome “Foro Italico”, 00135 Rome, Italy; 3 Division of Pediatric Gastroenterology and Nutrition, Mass General for Children and Harvard Medical School, Boston, MA 02114, USA; 4 Laboratório Alerta, Departamento de Medicina Interna, Divisão de Doenças Infecciosas, Escola Paulista de Medicina (EPM), Universidade Federal de São Paulo (UNIFESP), São Paulo 04021-001, SP, Brazil; 5 Department of Nutrition, Harvard T.H. Chan School of Public Health, Boston, MA 02115, USA; 6 European Biomedical Research Institute of Salerno, 84121 Salerno, Italy

**Keywords:** gut microbiome, 16S metabarcoding, shotgun metagenomics, elite athletes, sequencing methodology, microbiome profiling, exercise science

## Abstract

The growing interest in the role of the gut microbiome in athletic performance has led to the application of various sequencing technologies in this field. This review critically examines the sequencing methodologies used in microbiome studies on physical performance and sport, comparing their advantages, limitations, and applicability. In particular, the focus is on 16S metabarcoding and shotgun metagenomics, evaluating how these methodological approaches influence the interpretation of results in sports contexts. Close attention is directed toward technical challenges, methodological biases, and future perspectives, including emerging technologies and multi-omics approaches. This review aims to bridge the gap between methodological rigor and sports-specific applicability, providing evidence-based methodological guidance to support researchers in designing robust athlete microbiome studies and translating sequencing-derived findings into concrete applications for performance and sports health.

## Simple Summary

The technology used to investigate the gut microbiota of athletes determines not only the precision of the results but also the biological conclusions that can be drawn. This review compares two of the most widely used sequencing methods: a targeted approach that analyses a specific region of bacterial genetic material to identify which microorganisms are present and a broader approach that sequences the entire genetic material of a sample, providing information not only on microbial identity but also on the functional activity of those microorganisms within the host. Each approach offers distinct advantages and limitations in terms of cost, taxonomic resolution, and the biological insights it can provide. For researchers working with elite athletes, selecting an inappropriate sequencing method may result in the loss of critical information regarding microorganisms that directly influence performance. Practical, evidence-based guidance is therefore provided to help sports scientists select the most appropriate sequencing strategy for their research objectives, ultimately contributing to the development of personalized nutritional and recovery interventions for athletes.

## 1. Introduction

The human gut microbiota, composed of trillions of microorganisms, has emerged as a key player in influencing various aspects of human health, including athletic performance [1]. Physical exercise influences intestinal physiology through multiple mechanisms, including changes in blood flow, motility, and intestinal permeability that, in turn, modulate the microbiota [2]. Recent studies have demonstrated that physical exercise can modulate the composition and functionality of the gut microbiota, while the microbiota itself can influence recovery capacity, the inflammatory response, and energy metabolism in athletes [3,4]. In athletes, the microbiota plays a role in performance and health, as it can modulate training adaptations and respond to external stimuli [5]. Strenuous and prolonged training can induce chronic inflammation and intestinal dysbiosis [6,7,8,9,10,11,12,13]. A balanced microbiota can reduce inflammatory markers and reactive oxygen species (ROS) production, actively contributing to the management of potential suboptimal health effects [14].

Understanding the bidirectional relationship between microbiota and sport requires robust, accessible, and accurate analytical methodologies. Over the last few decades, microbial identification has shifted from phenotype-based approaches to genome-based analyses, leading to the adoption of the term “microbiome” to describe the total genomic content of the microbiota. Microbiome analysis has undergone rapid evolution in recent decades, transitioning from culture-dependent methods that identified only a limited fraction of intestinal microorganisms to advanced molecular techniques based on DNA sequencing. This transition has revolutionized the ability to characterize complex microbial communities, enabling the identification of thousands of previously unculturable species and the understanding of their metabolic functions, including in sports contexts [15]. This progress has been accelerated by the significant reduction in sequencing costs over the past two decades. According to the National Human Genome Research Institute (NHGRI), the cost of human whole-genome sequencing (WGS) has decreased from over $100 million in 2001 to just a few hundred dollars today for research-grade sequencing, thereby making high-throughput sequencing widely accessible and fueling the exponential growth of microbiome research [16]. However, the growing availability of different sequencing technologies has introduced new methodological challenges that require in-depth critical analysis. For this reason, recent guidelines have established standardized protocols for the collection, preservation, and analytical processing of gut microbiota samples in athletes, but a gap remains in understanding how different sequencing technologies influence study results and interpretations in the sports science context [17]. The choice of sequencing methodology can indeed significantly influence conclusions about microbiota–performance relationships, making a critical evaluation of the implications of each methodological approach necessary [15].

This review aims to compare the main sequencing technologies used in gut microbiome studies in sports contexts, analyze how different methodologies influence the interpretation of results, identify technical challenges and methodological biases that compromise study comparability, and support future guidelines for microbiome analysis in athletes.

## 2. High-Throughput Sequencing Technologies

Over the last few decades, sequencing technologies have evolved from a manual approach (Sanger sequencing) to several automated protocols, enabling the parallel reading of a large number of sequences [18]. Current DNA sequencers can be classified into two classes: instruments for next-generation sequencing (NGS), or second-generation sequencing, and instruments for third-generation sequencing (long-read sequencing). The two systems differ in many technical characteristics, starting with the sequencing chemistry adopted [19].

Illumina sequencing platforms are widely used for NGS applications. The technology is characterized by polymerase chain reaction (PCR) amplification: sequencing templates are immobilized on a specific flow cell, where they are clustered via solid-phase amplification, with densities reaching 10 million single-molecule clusters per square centimeter. Illumina sequencers are based on sequencing by synthesis (SBS) technology, which uses four fluorescently labeled deoxynucleoside triphosphates (dNTPs). During the sequencing reaction, the labeled dNTPs act as reversible terminators: after incorporation, the fluorescent dye is excited by a laser and detected, and then enzymatic cleavage allows for new incorporation. This technology is highly accurate, with an estimated error rate of at most 0.1% for the newest hardware released [20]. Illumina read length is around 300 base pairs (bp) (2 × 150 bp), and the newest sequencers are characterized by more than 20 billion reads passing the filter per flow cell. The run time for 300 bp amplicons ranges from 23 to 48 h, depending on the instrument and the type of flow cell.

An alternative method of NGS is represented by the Ion Torrent technology [21]. This approach is also characterized by PCR amplification: sequencing templates are bound to the surface of microscopic beads called ion sphere particles (ISPs) and amplified using emulsion PCR (emPCR). Statistically, each bead is bound to a single DNA fragment. At this point, amplified beads are loaded into microwells on a semiconductor chip known as a complementary metal-oxide semiconductor (CMOS). Subsequently, the sequencer employs SBS technology, but without the use of fluorescent dye. Nucleotides are sequentially added to the CMOS, and after polymerization, a hydrogen ion is released, changing the pH of the solution. The semiconductor chip converts these changes into a voltage signal. This technology is characterized by a short sequencing time. However, it can be less accurate for homopolymeric regions (sequences of repeating nucleotides) due to the release of a large amount of hydrogen ions. According to the manufacturer, the Ion GeneStudio™ S5 System can provide more than 100 million reads in under 12 h, with read lengths ranging from 200 to 600 bp [22].

Third-generation sequencing does not require a DNA amplification step, and for this reason, protocols require less time. Pacific Biosciences (PacBio) sequencers employ single-molecule real-time (SMRT) technology, which analyses individual molecules of DNA as they are synthesized [23]. The key element of the protocol is the adoption of a high-processivity DNA polymerase. The starting amount of DNA template is over 500 nanograms (ng) for each sequencing chip. The reaction is carried out on a specific chip called the SMRT cell. Each chip contains 150,000 ZeroMode Wave Guides (ZMW). Starting from a fragmented DNA, specific single-strand hairpin adapters are used to circularize the template, generating a circular DNA molecule known as the SMRTbell. Every SMRTbell diffuses in a single ZMW, where a polymerase immobilized at the bottom initiates synthesis, incorporating fluorescently labeled nucleotides. After excitation, specific light emissions are detected for the different nucleotides. This technology can be applied to continuous long reads (CLRs) for DNA fragments of more than 50 kilobase pairs (Kb). In the CLR mode, read accuracy is approximately 90%. To reduce the error rate, the sequencer can operate in circular consensus sequencing (CCS) mode, where the polymerase performs multiple synthesis passages on the same region to create a consensus sequence. The CCS mode, often referred to as high-fidelity (HiFi) sequencing, achieves single-molecule consensus accuracy of approximately 99.8%, representing a substantial improvement over CLR accuracy and matching or exceeding the ability of short-read NGS platforms to detect single-nucleotide variants (SNV) [24]. This level of accuracy enables reliable variant detection and complete genome assembly, albeit with the constraint of being applicable to DNA fragments with an average length of approximately 15 Kb [24].

Over the last decade, a new third-generation sequencing technology has become available through Oxford Nanopore Technologies (ONT) sequencers [25]. The technology is based on the adoption of flow cells, which contain an array of protein pores, or “nanopores”, embedded in an electrically resistant polymer membrane. A MinION flow cell contains 512 channels with 4 nanopores in each channel, for a total of 2048 nanopores. The membrane is immersed in an electrolyte solution, and a constant voltage is applied to produce an ionic current through the nanopores. DNA or RNA molecules, attached to specific motor proteins through an adapter, are loaded into the solution. The voltage drives the DNA molecule through a nanopore at a rate of approximately 400 bp per second. The passage of a nucleotide disturbs ion flows through the nanopore, and an electrical signal trace is recorded. The unique characteristics of each base cause a distinct ionic current perturbation, which is recognized by software such as MinKNOW. Specific ultra-long protocols can allow reads up to 2 megabase pairs (Mbp). Since the beginning of ONT sequencer commercialization, this technology has been characterized by an elevated error rate (around 10%) [26]. In the last few years, significant efforts have led to a reduction in base-calling errors, enhancing the overall precision of nanopore sequencing. Recent comparative studies have shown an average quality of between 96.8% and 99.5%, significantly reducing the gap with NGS technologies [27,28].

## 3. Sequencing Methodologies in Sports Medicine

### 3.1. 16S Metabarcoding

The most widely used sequencing approach in studies of the athlete’s gut microbiota is 16S Ribosomal RNA (16S rRNA) gene sequencing, also known as 16S metabarcoding. This methodology is based on PCR amplification and subsequent sequencing of the hypervariable regions of the bacterial 16S rRNA gene, which contains both conserved and variable regions, thereby allowing for taxonomic identification [29]. The typical workflow includes microbial DNA extraction from fecal samples, PCR amplification of one or more hypervariable regions of the 16S rRNA gene (V1–V3, V3–V4, or full-length V1–V9 for long-read platforms), sequencing library preparation, sequencing, and bioinformatic analysis for taxonomic assignment of obtained sequences [30]. 16S rRNA sequencing has been widely used to characterize differences in microbial composition between athletes and non-athletes. For example, Clarke et al. employed this methodology to demonstrate that professional rugby athletes exhibited greater microbial diversity compared to sedentary controls, which was positively correlated with protein intake and creatine kinase levels [31]. Additionally, Petersen et al. used 16S rRNA sequencing to characterize the microbiota of professional cyclists, identifying specific microbial community characteristics associated with elite cycling, including a greater abundance of taxa involved in amino acid pathways [32].

**Figure 1:**
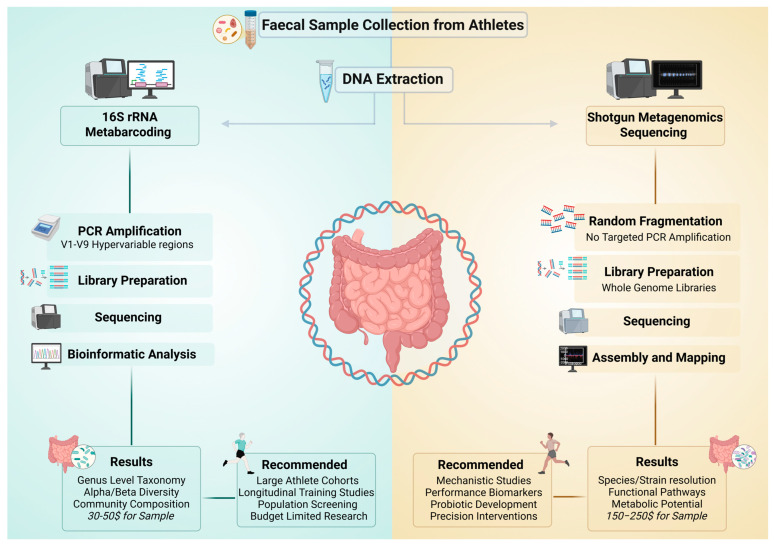
Methodological workflows for gut microbiota analysis in athletes: 16S rRNA metabarcoding and shotgun metagenomics sequencing.

The advantages of this methodology in the sports context include relatively low costs that enable the analysis of larger athlete cohorts, standardized protocols that facilitate comparability across studies, and greater bacterial specificity [33]. Moreover, it is sufficient for detecting differences in taxonomic composition at the genus level and is suitable for longitudinal studies monitoring microbiota changes during training or competition periods. The limitations of this methodology include reduced taxonomic resolution, which often does not allow identification at the species level, a lack of direct functional information on the microbiota, susceptibility to PCR-related biases, and a significant influence of hypervariable region selection on study results [33,34,35,36]. Controlled studies on artificial microbial populations have demonstrated how primer choice and extraction protocol can introduce significant biases in bacterial community representation [36]. These biases can be particularly critical for studies on elite athletes, where even small differences in microbial composition may have a significant impact on performance. Methodological validation through mock communities, therefore, represents a fundamental step in ensuring the accuracy of results in sports studies [14]. Nevertheless, the systematic use of mock communities and negative extraction controls remains uncommon in published athlete microbiome studies, limiting the ability to quantify technical noise and undermining the reproducibility of reported findings [14,17] (Figure 1).

### 3.2. Shotgun Metagenomic Sequencing

Shotgun metagenomic sequencing (SMS), an untargeted metagenomic approach, involves the direct sequencing of all DNA fragments extracted from a sample, without the use of PCR amplification to target specific genes. This methodology provides a more complete view of the microbial ecosystem, characterizing not only bacteria but also viruses, fungi, and other microorganisms [35]. The typical workflow includes total DNA extraction from the sample, random DNA fragmentation, sequencing library preparation, sequencing, de novo assembly, mapping to reference databases, annotation, and bioinformatic analysis for genomic reconstruction and functional inference. Currently, SMS can be performed at different depths: deep shotgun sequencing can generate more than 20 million reads per sample, while shallow shotgun sequencing is limited to approximately 5 million reads per sample.

The SMS approach has been used in more recent and in-depth studies on the gut microbiota of elite athletes. Scheiman et al. applied this methodology to identify *Veillonella atypica* in the microbiota of marathoners, demonstrating how *V. atypica* can improve endurance performance through lactate metabolism [37]. More recently, Fontana et al. used SMS to analyze 418 metagenomic datasets from athletes’ fecal samples, highlighting how competitive physical exercise is associated with modulation of the microbiota composition, with an increase in short-chain fatty acid (SCFA)-producing bacteria [15]. However, sequencing depth remains a critical factor, as shallow SMS via 0.5 million reads can provide limited information compared to deeper approaches [38]. The required sequencing depth varies significantly between methodologies. While 10,000–50,000 reads per sample may be sufficient for 16S metabarcoding, SMS requires at least 1–10 million reads for adequate characterization of the gut microbiota [39].

The advantages of the deep SMS methodology include superior taxonomic resolution, enabling identification at the species level. It is also possible to obtain insights into the genetic and functional potential of microbiota: the detection of specific microbial biomarkers associated with performance can support the reconstruction of metabolic pathways relevant to energy metabolism during exercise. In addition, PCR amplification biases are minimized, and the discovery of novel microbial taxa (which are not detectable with 16S metabarcoding approaches or shallow protocols) is supported [34,35,37,40]. The limitations of this methodology include a relatively high background noise (also due to potential DNA contamination) and a complex bioinformatic pipeline that requires intensive computational resources. In several situations, the higher costs can limit the size of analyzable cohorts, especially when a greater sequencing depth is required to capture low-abundance species [35,38,39,41]. Furthermore, the accuracy of SMS taxonomic classification remains dependent on the completeness and quality of reference databases, which represent an additional source of potential bias in characterizing the athlete’s gut microbiota (Figure 1).

## 4. Comparison Between 16S Metabarcoding and Shotgun Metagenomics Sequencing

An overview of key studies applying 16S metabarcoding and SMS to athlete gut microbiome research is provided in Table 1, illustrating the diversity of populations studied and the methodological heterogeneity of the existing literature.

### 4.1. Microbial Diversity and Performance Taxa Identification

Studies applying the two methodologies to gut microbiota samples have revealed significant discrepancies in microbial diversity assessment. SMS demonstrates superior detection, power, and precision compared to 16S metabarcoding [34]. A recent comparative analysis confirmed that SMS can identify a greater number of phyla, classes, orders, families, genera, and species in the same human fecal samples compared to 16S rRNA sequencing [56]. It is worth noting that 16S rRNA gene copy number varies among bacterial species, introducing an additional possible bias in microbial diversity analyses through 16S metabarcoding [57]. Louca et al. demonstrated that 16S rRNA gene copy numbers can be accurately predicted only for taxa with ≤15% sequence divergence from reference genomes, which explains less than 10% of the variance in predictive accuracy [58]. Another comparative study has demonstrated that SMS provides superior taxonomic resolution and more accurate quantification of microbial abundance, particularly for less abundant taxa, compared to 16S rRNA sequencing [59]. An essential consideration concerns the extent to which methodological choice can fundamentally alter biological conclusions in sports science research, not merely their precision. A pertinent example is provided by the genus *Veillonella*. Studies based on 16S metabarcoding report the relative abundance of *Veillonella*, but it was through SMS that Scheiman et al. identified *Veillonella atypica*, which appears to be able to convert exercise-induced lactate to propionate, thereby providing a mechanistic explanation for the improvement in exercise capacity [37]. Had the investigation relied solely on 16S metabarcoding, this genus-level association would have remained a descriptive finding with no actionable biological meaning. Similarly, the genus *Prevotella*, frequently identified as abundant in athletes, includes species with markedly different functional profiles, and SMS-based studies have revealed that sport-associated enrichment is predominantly driven by specific species involved in SCFA production [15,32]. These examples illustrate that the choice between 16S rRNA sequencing and SMS is not merely technical or economic but determines whether a study generates a hypothesis or tests a mechanism. Researchers in sports science should therefore orient their methodological selection according to the research objective, using 16S rRNA sequencing to identify patterns and generate hypotheses and SMS to validate, refine, and translate those hypotheses into biologically meaningful and potentially clinically applicable insights. These insights could be of considerable importance, as each sport discipline likely exhibits distinctive profiles and characteristics of the gut microbiota [50,53,55]. Methodological selection significantly influences research outcomes in microbiome studies across all fields, including sports medicine. For large-scale population studies, 16S rRNA sequencing can provide a cost-effective initial taxonomic screening method, suitable for identifying broad patterns [38]. Conversely, mechanistic studies that require functional pathway analysis necessitate SMS approaches, which provide a direct assessment of metabolic capabilities [38]. Considering that SMS currently represents the optimal solution in terms of precision and accuracy, but with relatively higher costs, a practical strategy could involve conducting an initial screening through 16S rRNA sequencing, aimed at the identification of broad taxonomic patterns and potential exercise-responsive taxa, followed by expanded analyses through SMS for functional validation and mechanistic investigation [38]. However, SMS data normalization requires more sophisticated bioinformatics approaches and extensive computational resources [60].

Overall, however, the available evidence reflects a field still characterized by substantial methodological heterogeneity that constrains cross-study comparability and the strength of conclusions that can be drawn regarding microbiota–performance relationships [3,15,17,37].

### 4.2. Result Reproducibility

The choice of sequencing methodology can significantly impact the reproducibility of results across studies. Methodological heterogeneity can represent a significant challenge for reviews and meta-analyses of athlete gut microbiota, thereby limiting the ability to identify microbial biomarkers associated with performance. Studies directly comparing 16S rRNA sequencing and SMS on the same stool samples have shown that 16S captures only part of the microbial community, focusing mainly on dominant taxa, whereas SMS provides a more comprehensive and detailed profile of microbial diversity [56]. Consequently, data obtained with the two methods cannot be directly compared across studies. Methodological choice also influences not only the detection of diversity but also the interpretation of evolutionary patterns within microbial communities [61]. Furthermore, inter-laboratory reproducibility in gut microbiome studies remains limited, as the systematic reporting of technical replicates is rarely adopted in athlete cohort studies, making it difficult to distinguish genuine biological variation from analytical noise [14,17].

### 4.3. Cost-Effectiveness Analysis

Despite its higher cost, SMS can offer better cost-effectiveness for studies focused on identifying specific functional mechanisms through which the microbiota influences performance [38]. Conversely, for large-scale epidemiological studies or longitudinal monitoring of athletes, 16S rRNA sequencing may represent the most practicable option [33]. Recent analyses have estimated that, although the sample cost of SMS is 2–3 times higher compared to 16S rRNA, the amount of functional information obtained can justify the investment when the objective is to understand specific mechanisms through which the microbiota influences exercise physiology and performance [35] (Table 2).

## 5. Technical Methods and Methodological Biases

### 5.1. Sample Collection and Preservation Protocols

The quality and representativeness of fecal samples are critical factors that can significantly influence results, regardless of the sequencing technology used. The life of an elite athlete is characterized by several events that can impact the stability of gut microbiota. In particular, extended travel periods, seasonal training changes, intense competition periods, and the use of supplements or medications should be considered when planning fecal sampling [41]. The interaction between diet and intensified training has a significant influence on microbiota composition, highlighting the importance of considering both factors in research studies [6]. Given the various difficulties related to fecal sample collection and preservation protocols, athletic populations present additional methodological challenges for scientists. Mancin et al. have described several standardization difficulties, including clinical data collection, fecal sample collection techniques (whole fresh stool vs. dry swab), timing and frequency of collection, preservation methods (−80 °C, −20 °C, +4 °C, or room temperature), technical sample preparation (DNA extraction), microbial assessment methods (target gene or whole genome), sequencing platforms, and statistical analysis approaches [17].

Some authors have suggested that variation in profiling techniques could act as a confounding variable, leading to divergent findings that are entirely due to laboratory techniques rather than treatment, and highlighting that many studies may be underpowered and fail to control for important athlete-specific confounding variables [3]. Furthermore, exercise-induced physiological changes could further complicate fecal collection protocols. Exercise intensity exceeding 70% of maximum oxygen consumption (VO_2_max) could delay gastric emptying [62]. Additionally, moderate-intensity aerobic exercise lasting approximately 2 h appears to represent the threshold beyond which relevant gastrointestinal alterations occur, with stress-induced at 60% VO_2_max identified as the critical point for the onset of such disturbances, regardless of training status [63].

Methodological biases may also depend on the preservation methods used for fecal samples, where flash-frozen samples showed higher proportions of *Actinobacteria* proteins, while preservation with RNAlater led to increases in *Bacteroidetes* protein proportions [64]. The *Firmicutes*/*Bacteroidetes* ratio, analyzed through the 16S rRNA gene, appears to be modified by sample freezing, where frozen samples seem to present higher values compared to fresh samples [65]. Moreover, DNA extraction methodology probably represents the most significant source of variation in gut microbiome studies, especially for amplicon sequencing [66]. Recent metagenomic validation studies have demonstrated differences in preservation efficacy between commercial and in-house systems for sampling the gut microbiota. Ambient temperature drying of swabs appears to offer good performance in terms of both technical and compositional reproducibility, as well as sample stability, making it potentially suitable for contexts with travel constraints [67]. To address these preservation-related challenges, various collection and storage systems have been systematically evaluated. In-house preservation methods offer potential cost-effective alternatives, with the nucleic acid preservation (NAP) buffer demonstrating superior preservation qualities compared to RNAlater and DNA/RNA Shield in direct comparisons for preserving bacterial diversity at room temperature for up to 10 days [68]. Camacho-Sanchez et al. demonstrated, using non-human fecal samples, that NAP buffer (0.019 M EDTA, 0.018 M sodium citrate tribasic, 3.8 M ammonium sulfate, pH 5.2) yielded DNA concentrations 1.3 times higher than 95% ethanol and significantly higher than cryopreserved samples after 7 weeks at room temperature [69].

In contrast, alternative cost-effective methods have been investigated, including dimethyl sulfoxide, EDTA, and NaCl solution (DESS) (20% dimethyl sulfoxide (DMSO), 0.25 M EDTA, saturated NaCl, pH 8.0), which appears to maintain 96% compositional similarity to frozen samples, and 95% ethanol-based preservation, which provides effective preservation for up to 8 weeks at room temperature [70]. While in-house and alternative preservation methods offer useful options for specific contexts, practical guidance for the design of athlete microbiome studies should prioritize validated, standardized solutions. When immediate freezing at −80 °C is not feasible, as is frequently the case during training camps, competition travel, or multi-site data collection, commercial room-temperature stabilization methods, including OMNIgene-GUT (DNA Genotek, Ottawa, ON, Canada), DNA/RNA Shield (Zymo Research, Irvine, CA, USA), and swab-based active drying systems, represent broadly validated and practical alternatives, demonstrating high compositional reproducibility compared to flash-frozen samples across shotgun metagenomic workflows [67]. Researchers should pre-select and validate their preservation method prior to study initiation and apply it consistently across all sampling time points to minimize preservation-induced variability as a confounding factor.

Given the substantial heterogeneity in microbiome assessment techniques, standardization of these methodological elements is essential. Only through consistent approaches can more reliable inferences be drawn and meaningful comparisons facilitated, both within and across athlete cohorts in sports microbiome research [17,71].

### 5.2. DNA Extraction and Sequencing Protocols

Microbial DNA extraction protocols differ considerably between laboratories and represent the most significant source of methodological bias in gut microbiome studies [72]. In athletic populations, standardization of extraction protocols becomes particularly critical due to the specific characteristics of athlete samples and the performance relevance of specific bacterial taxa that exhibit differential extraction sensitivity [17]. Commercial extraction kits appear to influence human microbiome research through multiple relevant parameters. Lim et al. demonstrated that QIAamp PowerFecal Pro showed comparable performance to the QIAamp DNA Stool Mini protocol with bead-beating and superior performance compared to the version without bead-beating [73]. Specifically, DNA purity, as calculated by the A260/A280 ratio, is optimal with the first kit [73]. In another recent comparative study of four extraction kits, including QIAamp PowerFecal Pro, differences in DNA quantity and quality did not substantially influence microbiota composition and diversity in fecal samples, while all kits demonstrated low sensitivity for low-biomass samples [74]. Furthermore, multi-kit comparisons suggest that these kits likely produce high-quality and high-integrity DNA suitable for 16S metabarcoding [75]. Mechanical versus chemical lysis methodologies can significantly influence the detection of bacterial taxa. The International Human Microbiome Standards (IHMS) Protocol Q represents the current standard practice, which includes mechanical lysis with 0.1 mm zirconia beads [72,76]. Nevertheless, some authors have suggested that protocols incorporating bead-beating should be characterized by an improved capability to lyse difficult bacteria (such as *Gram-positive* bacteria), including taxa such as *Firmicutes* and *Actinobacteria*, without significantly altering the ratios between major phyla [77,78].

16S rRNA gene sequencing is subject to biases due to PCR amplification, including preferential amplification of some taxa over others, chimera formation, variable primer coverage for different taxonomic groups, and variable copy number of the 16S rRNA gene between different species [33]. However, an important advantage of 16S rRNA gene sequencing remains the targeted amplification of bacterial genes, which limits human DNA contamination compared to SMS. The adoption of primers specific for several variable regions of the 16S rRNA gene, such as the V3–V4 and V1–V2 primers, strongly reduces background noise without affecting taxonomic identification [79]. In contrast, in SMS, human DNA contamination can reach high levels, especially in clinical samples, thereby reducing the sequencing depth available for microbial analysis and necessitating increased sequencing to achieve equivalent microbial coverage [80]. Fecal samples analyzed via 16S metabarcoding typically contain less than 10% human DNA contamination, making this approach more suitable for studies with limited sequencing budgets or samples with anticipated host DNA interference [79,81,82].

### 5.3. Bioinformatic Pipelines and Analytical Approaches

The choice of a reference database for taxonomic assignment can significantly influence the interpretation of results, particularly for less studied taxa. The most widely used databases for 16S metabarcoding are Greengenes, SILVA, and the Ribosomal Database Project (RDP). In contrast, the National Center for Biotechnology Information (NCBI) Reference Sequence Database (RefSeq) remains the most adopted database for SMS. Recently, authors have highlighted critical points in SMS when adopting RefSeq, suggesting the need for more accurate reference databases [83].

Different bioinformatic pipelines, such as QIIME, Mothur, and DADA2 for 16S rRNA, and MetaPhlAn, Kraken, and HUMAnN for SMS, employ various algorithms for taxonomic classification and functional inference, contributing to result variability between studies [84,85,86]. The integration of data from different sequencing methodologies represents a significant challenge for the more comprehensive characterization of an athlete’s microbiome. Complete methodological reviews provide essential guidance for taxonomic classification and metagenomic assembly, particularly for detailed studies of athletes’ gut microbiome [87]. Bebawy et al. highlighted how the choice of classifier software in SMS can impact the accuracy of microbial classification, prompting discussion of pipeline standardization [88].

### 5.4. Athlete-Specific Confounding Variables

In addition to technical and analytical sources of bias, athlete microbiome studies are subject to a set of biological and behavioral confounding variables that are complex to evaluate and manage simultaneously. Diet represents one of the most influential modifiable confounding factors, as macronutrient distribution can substantially alter gut microbial composition within hours or days [3,4,6]. However, in many athlete microbiome studies, given the inherent logistical difficulties, dietary intake in the days preceding fecal sampling is not standardized, and when assessed, it is often evaluated using retrospective instruments such as food diaries or food frequency questionnaires, which are subject to memory bias and under-reporting [3,17]. This heterogeneity in the management of the dietary variable makes it more difficult to disentangle microbiota–exercise associations from effects attributable to nutritional intake.

Nutraceutical supplementation and medication use represent additional relevant confounding factors. For example, antibiotics, non-steroidal anti-inflammatory drugs (NSAIDs), proton pump inhibitors, oral contraceptives, dietary fiber, prebiotics, probiotics, postbiotics, synbiotics, melatonin, and protein supplements are widely used in athletic populations, and each has been shown to affect gut microbial composition [3,6,17]. Although many studies apply exclusion criteria for antibiotic use within a defined washout period, objective verification through medication logs or biomarker screening is rarely reported, introducing uncontrolled variability that may systematically bias comparisons between athlete cohorts.

Training load at the time of sampling, including intensity, volume, frequency, and cumulative fatigue, is typically described qualitatively rather than quantified using validated metrics [4,17]. This makes it considerably more difficult to distinguish microbiota patterns reflecting long-term training adaptation from those attributable to acute physiological stress responses.

Given the logistical constraints inherent to research with elite athlete populations, complete simultaneous control of all confounding variables is rarely feasible. A pragmatic, tiered approach is therefore recommended, prioritizing systematic training load documentation and short-term dietary recording as minimum requirements, with more comprehensive dietary standardization and biochemical monitoring reserved for mechanistic study designs [17].

## 6. New Frontiers in Athlete Microbiota Sequencing

Third-generation long-read sequencing platforms, including Oxford Nanopore and PacBio, are emerging as powerful tools for high-resolution characterization of the microbiome. These technologies enable full-length 16S rRNA gene sequencing with enhanced taxonomic resolution, improved microbial genome assembly, and comprehensive characterization of mobile genetic elements [89,90,91]. Although applications in sports science remain limited, recent studies have employed advanced metagenomic approaches to reconstruct complete microbial genomes from athletes’ gut microbiota [46]. Integrating metagenomic and metatranscriptomic data with clinical and physiological parameters, including performance metrics, oxidative stress and inflammation biomarkers, body composition, and energy metabolism, can provide a dynamic, functional view of the microbiome in response to training, competition, and dietary periodization [4,7,15,51]. The lactate-to-propionate conversion mediated by *Veillonella atypica* exemplifies the translational value of integrating metagenomic data with physiological performance metrics [37]. The progressive refinement of these sequencing approaches, combined with the development of sport-specific normative databases for microbial composition and function, may ultimately support the translation of microbiome findings into individualized nutritional and recovery strategies for athletes.

To maximize the translational value of these approaches, standardization of microbiome analysis from sample collection to bioinformatic processing is essential for improving reproducibility and cross-study comparability [17,92]. Key requirements include systematic sample collection and preservation protocols, optimized DNA extraction methods to ensure comprehensive microbial representation, and consistent bioinformatic pipelines. Experimental design should incorporate appropriate control groups or utilize a within-subject longitudinal design, accounting for sport-specific confounders, including nutritional intake, supplement and medication use, psychological stress, host biomarkers, and sampling timing relative to training and competition periods [48,49,93]. Detailed metadata collection is equally critical, as sport type and sport-specific dietary patterns produce distinctive microbial signatures that may inform personalized nutritional interventions [51,52,94].

The sequencing approach should align with the study objectives. 16S metabarcoding is suitable for large-scale epidemiological studies, longitudinal monitoring across training seasons, initial taxonomic screening, and resource-limited settings [43,54]. Conversely, SMS is preferable for in-depth characterization of microbial biomarkers, identification of functionally relevant metabolic pathways, discovery of athlete-specific probiotic strains, and mechanistic investigations, such as fecal microbiota transplantation studies [15,44,45,47,95,96]. However, high costs and technical challenges currently limit the widespread adoption of these advanced sequencing solutions.

**Figure 2:**
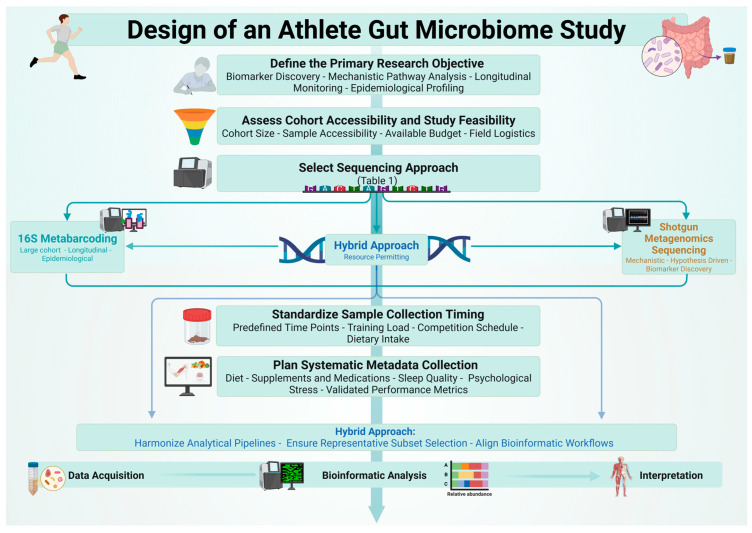
Decision framework for selecting sequencing methodologies in athlete gut microbiome research. Key decision nodes include research objective, cohort accessibility, available budget, and metadata requirements, guiding researchers toward 16S metabarcoding, shotgun metagenomics sequencing, or a hybrid approach. Data acquisition, bioinformatic analysis, and interpretation proceed regardless of the selected sequencing strategy.

To support researchers in selecting the most appropriate sequencing strategy, a structured decision framework is proposed (Figure 2). Methodological selection should be primarily driven by the research question, with a secondary consideration of practical constraints, including cohort size, available budget, and sample collection logistics.

## 7. Conclusions

Research on the gut microbiome in relation to athletic performance represents a rapidly evolving field, although it remains characterized by considerable methodological heterogeneity. Advances in sequencing technologies have significantly enhanced the ability to characterize the athlete’s microbiome. Nonetheless, methodological choices critically influence not only the results generated but also the inferences that can be reliably drawn. Future investigations are expected to benefit from integrated approaches combining multiple sequencing modalities with multi-omics analyses. Specifically, the integration of SMS with metatranscriptomic, metaproteomic, metabolomic, and lipidomic data may provide a more comprehensive functional characterization of microbiota–performance interactions. In contrast, 16S metabarcoding, although less comprehensive functionally, remains a valid and practical approach for repeated-measures or longitudinal study designs in sports science research, particularly when combined with physiological data.

Optimization and standardization of sequencing methodologies represent fundamental prerequisites for the development of practical applications. Potential applications include identifying microbial biomarkers predictive of training adaptation or susceptibility to overreaching and overtraining syndromes, as well as formulating targeted nutritional and supplementation strategies.

The integration of microbiome sequencing with complementary sport-specific and clinical data may further support the development of individualized training and nutritional programs, optimized recovery protocols, and preventive strategies for exercise-induced gastrointestinal issues. The choice of sequencing methodologies should be guided not solely by technical and economic factors but also by the relevance of the data obtained to specific research objectives and their potential clinical applicability. The methodological recommendations proposed in this review aim to provide researchers in sports science and medicine with evidence-based guidance to optimize study design and facilitate the translation of research findings into sport-relevant interventions. A critical, unresolved challenge is the fragmentation of athlete microbiome data across studies that differ in sequencing platforms, bioinformatic pipelines, and metadata structures, rendering cross-study synthesis and meta-analyses methodologically unreliable. The development of standardized, openly accessible reference databases specifically curated for athlete gut microbiome data, analogous to existing curated metagenomic resources for clinical populations, could represent a strategic priority for the field. Such infrastructure would enable robust meta-analyses, facilitate the identification of sport-specific microbial signatures, and accelerate the translation of microbiome research into applied practice.

Ultimately, the methodological rigor advocated in this review aims to advance sport microbiome research beyond descriptive associations toward a causal understanding capable of informing targeted prebiotic, probiotic, postbiotic, and synbiotic interventions, personalized nutritional strategies, and evidence-based recovery protocols. Achieving this goal requires not only methodological standardization but also a deliberate integration of microbiome science with exercise physiology, sports nutrition, and clinical practice.

## Figures and Tables

**Table 1:** Sequencing methodologies and study populations in gut microbiota research on elite and high-level athletes.

Study	Population	Sample Size	Sequencing Technology
Carlone et al. (2025) [5]	Elite Volleyball Players (Four timepoints)	7	16S ribosomal RNA amplicon sequencing (V2–V9, Ion GeneStudio S5, Ion AmpliSeq)
Charlesson et al. (2025) [42]	High-Level Rowers	23	16S ribosomal RNA amplicon sequencing (V4, Illumina MiSeq) and targeted fecal SCFA analysis
Henningsen et al. (2025) [43]	High-Level Ultra-Marathon Runners	13	16S ribosomal RNA amplicon sequencing (V3–V4, Illumina MiSeq)
Martin et al. (2025) [44]	Elite Soccer Players, Elite Cyclists and Non-Athletes	50	Shotgun metagenomics (MGI DNBseq-G400) and targeted fecal metabolomics
Aya et al. (2025) [45]	Elite Weightlifters versus Elite Cyclists	29	Shotgun metagenomics (Illumina HiSeq 2500), metabolomics and lipidomics
Wosinska et al. (2024) [46]	Elite Athletes across Multiple Sports, Moderate Athletes and Sedentary Controls	682	Shotgun metagenomics (re-analysis of publicly available datasets, short-read taxonomic profiling and metagenome-assembled genome recovery)
Fu et al. (2024) [47]	Elite Wrestlers (High versus Low-Performance)	12	16S ribosomal RNA amplicon sequencing (V3–V4, Illumina NovaSeq 6000) and untargeted metabolomics of fecal and urine
Fontana et al. (2023) [15]	Elite Athletes across Multiple Sports, Moderate Athletes and Sedentary Controls	418	Shotgun metagenomics (Re-analysis of publicly available datasets, METAnnotatorX2 pipeline)
Akazawa et al. (2023) [48]	Elite Athletes across Multiple Sports (Transition versus Preparation Phase)	94	16S ribosomal RNA amplicon sequencing (V3–V4, Illumina MiSeq)
Oliveira et al. (2022) [49]	Elite Football Players (Pre versus Post-tournament)	17	16S ribosomal RNA amplicon sequencing (V3–V4, Ion Torrent PGM)
O’Donovan et al. (2020) [50]	Elite Athletes across Multiple Sports	37	Shotgun metagenomics (Illumina NextSeq) and Metabolomics
Scheiman et al. (2019) [37]	Marathon Runners and Sedentary Controls; Elite Ultra-Marathoners and Olympic Trial Rowers	112	16S ribosomal RNA amplicon sequencing (V4, Illumina MiSeq) and shotgun metagenomics (Illumina HiSeq 2500)
Jang et al. (2019) [51]	Bodybuilders, Elite Runners, and Sedentary Controls	45	16S ribosomal RNA amplicon sequencing (V3–V4, Illumina MiSeq)
Liang et al. (2019) [52]	Professional Martial Arts Athletes (Higher versus Lower-Level)	28	16S ribosomal RNA amplicon sequencing (V3–V4, Illumina HiSeq 2500)
Murtaza et al. (2019) [6]	Elite Race Walkers (Baseline versus Post Dietary Interventions)	21	16S ribosomal RNA amplicon sequencing (V6–V8, Illumina MiSeq)
Keohane et al. (2019) [53]	Elite Rowers (Transatlantic Rowing Race, 33 days)	4	Shotgun metagenomics (Illumina NextSeq 550)
Zhao et al. (2018) [54]	Recreational Half-Marathon Runners (Pre- and Post-Race)	20	16S ribosomal RNA amplicon sequencing (V3–V4, Illumina HiSeq) and untargeted fecal metabolomics
Barton et al. (2018) [55]	Elite Rugby Players versus Sedentary Controls	86	Shotgun metagenomics (Illumina HiSeq 2500) and metabolomics
Petersen et al. (2017) [32]	Professional versus Amateur Competitive Cyclists	33	Shotgun metagenomics and metatranscriptomics (Illumina NextSeq/HiSeq); 16S ribosomal RNA amplicon sequencing (V1–V3, Illumina MiSeq)
Clarke et al. (2014) [31]	Elite Rugby Players versus Sedentary Controls	86	16S ribosomal RNA (V4, Roche 454 GS FLX pyrosequencing)

SCFAs = short-chain fatty acids.

**Table 2:** Comparison of 16S rRNA metabarcoding and shotgun metagenomic sequencing in studies on athletes’ gut microbiota.

Parameter	16S rRNA Metabarcoding	Shotgun Metagenomics Sequencing
Cost for sample	$30–50	$150–250
Taxonomic resolution	Genus level	Species and strain level
Functional information	Limited (inferred)	Direct pathway analysis
PCR amplification bias	Present	Absent
Host DNA contamination	Scarcely affected	Highly affected
Sample throughput capacity	High (large cohorts)	Limited (smaller cohorts)
Computational requirements	Standard pipelines	Intensive computing
Novel taxa detection	Limited to existing databases	Comprehensive discovery
Data storage requirements	Low (GB)	High (TB)
Expertise required	Moderate	Advanced bioinformatics
Performance biomarkers	Genus-level associations	Mechanistic insights
**Recommended Applications**		
Epidemiological studies	Suitable but limited to genus level	Limited by cost
Longitudinal monitoring	Suitable	Limited by cost
Mechanistic studies	Limited resolution	Suitable
Probiotic development	Limited	Suitable
Clinical translation	Population studies	Precision medicine
**Recommended Use**	Large-scale epidemiological studies; Longitudinal monitoring across training seasons; Initial taxonomic screening; Repeated-measures designs in field conditions	In-depth characterization of performance-related microbial biomarkers; Functional metabolic pathway analysis; Discovery of athlete-specific probiotic candidates; Mechanistic investigations; Multi-omics integration

(GB = Gigabytes; TB = Terabytes).

## Data Availability

No new data were created or analyzed in this study.

## Abbreviations

The following abbreviations are used in this manuscript: **ROS**, Reactive Oxygen Species; **WGS**, Whole-Genome Sequencing; **NHGRI**, National Human Genome Research Institute; **NGS**, Next-Generation Sequencing; **PCR**, Polymerase Chain Reaction; **SBS**, Sequencing by Synthesis; **dNTPs**, Deoxynucleoside Triphosphates; **bp**, Base Pairs; **ISPs**, Ion Sphere Particles; **emPCR**, Emulsion Polymerase Chain Reaction; **CMOS**, Complementary Metal-Oxide Semiconductor; **PacBio**, Pacific Biosciences; **SMRT**, Single-Molecule Real-Time; **ng**, Nanograms; **ZMW**, ZeroMode Wave Guides; **CLRs**, Continuous Long Reads; **Kb**, Kilobase Pairs; **CCS**, Circular Consensus Sequencing; **HiFi**, High Fidelity; **SNV**, Single-Nucleotide Variants; **ONT**, Oxford Nanopore Technologies; **Mbp**, Megabase pairs; **16S rRNA**, 16S Ribosomal RNA; **SMS**, Shotgun Metagenomic Sequencing; **SCFAs**, Short-Chain Fatty Acids; **GB**, Gigabytes; **TB**, Terabytes; **VO_2_max**, Maximum Oxygen Consumption; **NAP**, Nucleic Acid Preservation; **EDTA**, Ethylenediaminetetraacetic acid; **DESS**, Dimethyl Sulfoxide, **EDTA**, and NaCl Solution; **DMSO**, Dimethyl Sulfoxide; **IHMS**, International Human Microbiome Standards; **RDP**, Ribosomal Database Project; **NCBI**, National Center for Biotechnology Information; **RefSeq**, Reference Sequence Database; **NSAIDs**, Non-Steroidal Anti-Inflammatory Drugs.
